# Brazilian air traffic controllers exhibit excessive sleepiness

**DOI:** 10.1590/S1980-57642011DN05030009

**Published:** 2011

**Authors:** Valdenilson Ribeiro Ribas, Cláudia Ângela Vilela de Almeida, Hugo André de Lima Martins, Carlos Frederico de Oliveira Alves, Marcos José Pinheiro Cândido Alves, Severino Marcos de Oliveira Carneiro, Valéria Ribeiro Ribas, Carlos Augusto Carvalho de Vasconcelos, Everton Botelho Sougey, Raul Manhães de Castro

**Affiliations:** 1 Doctor in Neuropsychiatry; 2 Doctor in Biological Sciences; 3 Master in Neurology; 4 Master in Clinical Psychology; 5 Specialist in Surgery and Maxillofacial; 6 Expert in Public Health; 7 Master in Neuroscience; 8 Doctor in Neuropsychiatry; 9 Doctor in Mental Health; 10 Doctor in Experimental Pharmacology

**Keywords:** somnolence, sleep latency, air traffic controllers

## Abstract

Excessive sleepiness (ES) is an increased tendency to initiate involuntary sleep for naps at inappropriate times.

**Objective:**

The objective of this study was to assess ES in air traffic controllers (ATCo).

**Methods:**

45 flight protection professionals were evaluated, comprising 30 ATCo, subdivided into ATCo with ten or more years in the profession (ATCo≥10, n=15) and ATCo with less than ten years in the profession (ATCo<10, n=15) and 15 aeronautical information services operators (AIS), subdivided into AIS with ten years or more in the profession (AIS≥10, n=8) and AIS with less than ten years in the profession (AIS<10, n=7), who were included as the control group. The Epworth Sleepiness Scale and Maintenance of Wakefulness Test were used for evaluating subjective and objective excessive sleepiness. Kruskal-Wallis was used for ES and Mann-Whitney for sleep latency (SL), collection time in minutes (mins), and expressed as Median (Minimum-Maximum), p<0.05.

**Results:**

ATCo≥10 12 (6-14) mins and ATCo<10 10 (1-15) mins showed greater sleepiness compared to CONTROL1 7 (3-8) mins and CONTROL2 6 (4-6) mins, p=0.001*. A total of 77.27% of the ATCo and 16.67% of the AIS had an SL of less than 20 minutes. The ATCo presented an SL of 16.59 (3.25-40), lower than that of the AIS of 31.71 (10.63-40) mins, p<0.05*.

**Conclusion:**

Brazilian air traffic controllers exhibit excessive sleepiness.

## Abstract (pt)

A sonolência excessiva (SE) é a uma tendência aumentada de se iniciar o sono por cochilos involuntários em momentos inapropriados.

**Objetivo:**

O objetivo deste trabalho foi avaliar SE em controladores de trafego aéreo (CTA).

**Métodos:**

Foram avaliados 45 profissionais de proteção ao voo, sendo 30 CTA, subdivididos em CTA com dez ou mais anos na profissão (CTA≥10, n=15) e CTA com menos de dez anos na profissão (CTA<10, n=15) e 15 operadores de serviços de informações aeronáuticas (AIS), subdivididos em AIS com dez anos ou mais na profissão (AIS≥10, n=8) e AIS com menos de dez anos na profissão (AIS<10, n=7). A Escala de Sonolência de Epworth e o Teste de Manutenção da Vigília foram empregados para avaliação subjetiva e objetiva de sonolência excessiva. Utilizou-se: Kruskal-Wallis para os dados de SE e Mann-Whitney para a latência de sono. Tempo coletado em minutos (mins). Todos os dados foram expressos em mediana (mínimo-máximo), p<0,05).

**Resultados:**

CTA≥10 12 (6-14) mins e CTA<10 10 (1-15) mins apresentaram um aumento de sonolência, quando comparados ao CONTROLE1 7 (3-8) mins e ao CONTROLE2 6 (4-6) mins, p=0,001*. 77,27% dos CTA e 16,67% dos AIS apresentaram latência de sono abaixo de 20 minutos. Os CTA apresentaram uma latência de sono de 16,59 (3,25-40) mins abaixo dos AIS 31,71(10,63-40), p<0,05*.

**Conclusão:**

Controladores de tráfego aéreo apresentam sonolência excessiva.

## Introduction

Air traffic control is a profession that requires the constant action of its professionals due to the need to oversee all the flights on different schedules. In contrast to professions in which there are fixed hours of work such as from 8 a.m. to 4 p.m. this group of professionals works alternate shifts in order to attend the nightly demand of aviation.^1^

Worldwide, this activity is performed based on three units of control, namely: Tower (TWR), Approach Control (APP) and Area Control Center (ACC), which as a convention, including in Brazil, are acronyms from the English language. The controllers from the Tower are responsible for traffic during landing and take-off situations, and also for the movements of people and vehicles in the maneuvering area, and for monitoring the tracks and roads used for local circulation. In terms of vertical division, the tower has jurisdiction over all traffic flying at altitudes up to 2,000 feet.^2^

Approach Control (APP) is the entity responsible for the intermediary phase of the flight. In large capitals, areas are usually mapped out into terminals (TMA) that consist of route letters and manuals available to the airmen. These areas cover a lateral approach to a distance of 40 nautical miles (NM) or slightly over. APP has jurisdiction over traffic that flies at between 2,000 and 14,000 feet.^3^

**Figure 1:**
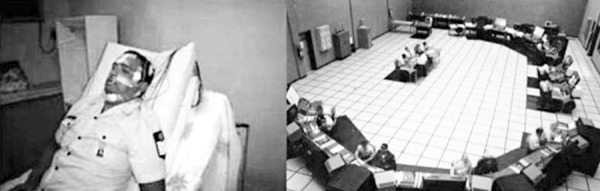
Air traffic controller undergoing the maintenance of wakefulness test (MWT) - to the right, Recife area control center (ACC).

The Area Control Center (ACC) usually controls a much larger air space than the above-mentioned organs. ACC Recife, for example, controls all aircraft in the whole Northeast of Brazil flying at 14,000 feet or above. The AIS, despite also having shift work, carries out a very different service to the air traffic controllers. AIS organizes publications that involve flight protection in the form of Aeronautics Command Instructions (ICAs), among them ICA 100-12, describing air traffic regulations for the whole of Brazil. The Area Control Center (ACC) (Figure 1), usually possess spaces with much broader jurisdiction than the above-mentioned organs. ACC Recife, for example, controls the aircraft in the whole Northeast of Brazil that are flying above 14,000 feet.^2^

Working in parallel with air traffic controllers (ATCo) are other professionals to protect the flight, including the aeronautical information service operators (AIS). The AIS organize publications that involve flight protection, such as Aeronautics Command Instructions (ICAs). Among these ICAs is ICA 100-12, which is related to the nationwide air traffic rules. Besides this type of activity, they also guide the pilots and the operational flight dispatchers (DOV) during the completion of flight plans and give them important notices about flight security called Notice to Airmen (NOTAM). These NOTAM contain information about restricted, dangerous or prohibited areas relating to air combat training by fighters from the Brazilian Air Force, Army and Navy, all occupying defined areas of Brazilian air space. Pilots will not be able to fly or overfly these zones and are obligated pursuant to ICA 100-12 to be aware of all NOTAMs before departure.^4^

This type of work, which is carried out during a phase of psychosomatic deactivation and resting periods, may cause fatigue in these individuals and compromise physical and/or mental performance after long periods of activity, provoking an inversion of biological rhythms and desynchronization of sleep-vigil states and circadian cycle (day/night).^5^

Typically, the quality of life for most individuals that work night shifts is affected due to sleep disorders and other alterations, such as cardiovascular, mood, anxiety, digestive and neuropsychological disorders. In addition,, reduced motivation for working, feeling of being ostracized, disturbance in family life, reduction of auditory and visual stimuli, etc.^6^

Besides the problems caused by the shift inversion, air traffic controllers perform simultaneously in the course of their activities, complex and multiple tasks, such as controlling several aircraft at the same time, coordinating flights with adjacent organs and planning ahead, involving leveled aircraft separation and also climbing or descending. In this context, the concern over the need for higher concentration level comes to the fore in professionals that may be affected by fatigue.^7^

Air traffic controllers carry out an activity that involves stress and sleep compromise while also working in alternate shifts. Stress itself affects sleep quality and, in addition the shifts, seem to cause a reduction in the quantity and quality of sleep, especially deep or slow-wave sleep, also called non-REM (*non-Rapid Eye Movement* - NREM), when restoration of the immune system and increase in interleukin synthesis occur.^8^

Under normal conditions, an individual starts their sleep at stage I of NREM sleep, after a period of latency of up to 10 minutes. A very low latency for the beginning of NREM sleep may occur in individuals that suffer from sleep deprivation, or those that are too tired, and is a phenomenon also found in syndromes involving non-restorative sleep, such as respiratory disorders. After a few minutes at sleep stage I, there is a deepening to sleep II, when it becomes more difficult for the individual to be awoken.^9^

After about 90 minutes, the first REM sleep occurs, which has a short duration (5 to 10 minutes), completing the first cycle of NREM-REM sleep. The exit from REM sleep may take place with the inclusion of micro-arousals (3 to 15 seconds), without complete awakening, changing to stage I NREM and then the stage II of NREM sleep, or passing directly to this last stage and deepening again to stage III and IV. A total of 5 to 6 cycles of NREM-REM sleep thus repeat over an 8-hour sleeping period.^10^

Among adults, daily sleep needs vary from 5 to 8 hours on average. Most adults do not feel completely recovered from their need to sleep if sleeping for less than 7 hours a day, although sociocultural demands usually lead them to sleeping less than their endogenous needs. Total sleep deprivation in one night causes a sleep debt phenomenon in the next two nights. Hence, there is a tendency for increased REM sleep proportions the night following deprivation, and an increase in NREM sleep on the second night, returning to the normal sleep architecture only on the third night.^11^

In this context, individuals that work at night such as air traffic controllers, who often change shifts without a fixed rota, may show disturbed sleep architecture besides symptoms of tiredness, irritability, intellectual alterations and excessive daily sleepiness alternating with insomnia.^10^ The hypothesis of this study was that air traffic controllers have excessive sleepiness. Thus, the objective of the study was to evaluate subjective and objective symptoms of excessive sleepiness in air traffic controllers from Recife area control center (ACC).

## Methods

This study was approved by the Ethics Committee of the Federal University of Pernambuco on November 1st, 2006. Before data collection, all subjects evaluated gave their written informed consent.

### Subjects

A total of 45 flight protection professionals were evaluated, comprising 30 air traffic controllers (ATCo) and 15 aeronautical information service operators (AIS) who were included as the control group. Notwithstanding shift work, the aeronautical information operators (AIS) perform a different role to that of controllers. The specification of this function will be outlined below. The professionals work at the Third Integrated Center of Air Defense and Air Traffic Control (CINDACTA III) and the Aeronautical Command (COMAER) in Recife/PE, Brazil. More specifically, the air traffic controllers were from the area control Center (ACC). The subjects were submitted to the evaluations at the CINDACTA III, under standard conditions at the beginning of their shifts at 08:00a.m in a building with central air-conditioner, maintaining a temperature of 22°±2°C. The professionals were informed about the test on the previous day and all subjects agreed to sleep at 08:00 p.m. the day before the tests. Male air traffic controllers were included in the research whereas inactive or female controllers were excluded because of their small number.

### Groups

The subjects were divided into four groups: two control groups comprising aeronautical information service operators AIS, subdivided into AIS male operators in the 30-45 year age bracket with ten years or more in the profession (AIS≥10, CONTROL 1), n=8; AIS male operators in the 18-29 year age bracket with less than ten years in the profession (AIS<10, CONTROL 2), n=7; a group of air traffic controllers ATCo with male controllers in the 30-45 year age bracket with ten years or more in the profession (ATCo≥10), n=15 and another comprising ATCo male operators in the 18-29 year age bracket with less than ten years in the profession (ATCo<10), n=15. All subjects held University degrees. The approach adopting a 10-year cut-off as a parameter for the data collection and group split was based on a previously conducted doctoral thesis.^19^ In the cited study, information was collected by questionnaire and included data such as headache, anxiety, depression, hypertension, infections and viruses, with results showing greater perseverance after ten years in the profession.

### Evaluation

Assessment was carried out in two steps:

- **1^st^ Step: Subjective Evaluation - Epworth Sleepiness Scale application (ESS)^12^**
  The Epworth Sleepiness Scale is a self-rated questionnaire with 8 questions, described in detail, relating to the chances of falling asleep or dozing off in each situation. These represent different situations but are still found in daily life.^20^
  The question is: what is the chance of falling asleep or dozing off in the following situations: Watching television? - Reading? - Lying down to rest in the afternoon? - At a public meeting? - Sitting after lunch? - Talking to someone? - In a car for more than an hour?
  The answers must be written rated from 0 to 3, with the following meanings: 0: would never doze, 1: slight chance of dozing, 2: moderate chance of dozing and 3: high chance of dozing.
- **2^nd^ Step: Objective Evaluation - Maintenance of Wakefulness Test (MWT) (Figure 1)**
  In this test, the subject must sit down in a chair or on a bed, leaning back slightly, in a silent dimly lit room with instructions to stay awake or not to sleep. The subject is not allowed to do other things to try and keep themselves awake. This includes actions such as singing or slapping their face. In order to monitor the wakefulness level with precision, sensors are placed on the head, face and chin. In other words, it is a specific electroencephalogram developed for studies related to sleep compromise. The test is performed in four sessions of 40 minutes. The average sleep latency test considered in the MWT is the arithmetic mean of the four sessions of 40 minutes. If the subject, despite having been instructed to stay awake, sleeps in less than 20 minutes, excessive sleepiness is diagnosed.
  Data collection was done in the morning at 8 a.m. at the beginning of each shift.

### Data analysis

The results found were analyzed using Kruskal-Wallis one way analysis of variance by ranks test, all pairwise multiple comparison procedures were performed by the Dunn’s method and data expressed in Median (Minimum and Maximum) for subjects’ data and by the Mann-Whitney test for objective data, with a significance level of p<0.05.

## Results

### Subjective evaluation of excessive sleepiness Epworth Sleepiness Scale (ESS)

**Figure 2:**
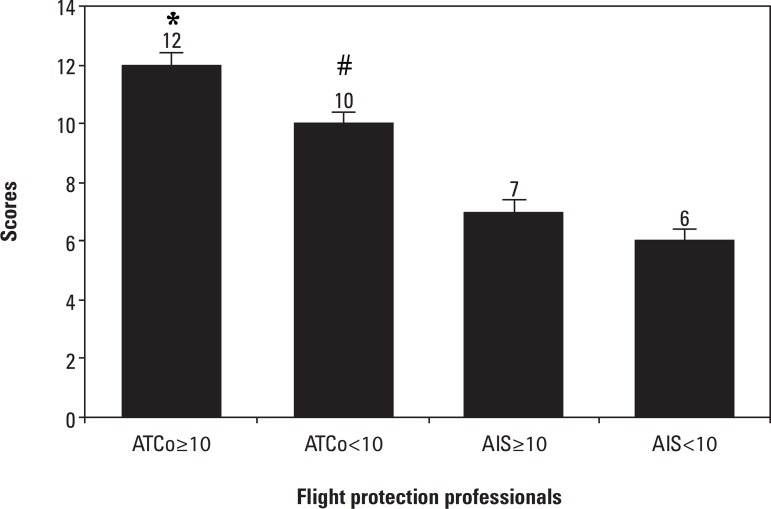
Assessment of excessive sleepiness in Air Traffic Controllers. According to the Epworth Sleepiness Scale, values above 9 indicate excessive sleepiness. The results found were analyzed by Kruskal-Wallis one way analysis of variance by ranks test, all pairwise multiple comparison procedures used Dunn’s method and data are expressed in Median (Minimum and Maximum) for subjects’ data, p≤0.05.

ATCo≥10 12 (6-14) and ATCo<10 10 (1-15) presented an increase in the subjective expression of sleepiness on the Epworth numerical scale of sleepiness (ESS)compared to the AIS operators AIS≥10, CONTROL1 7 (3-8), p=0.001* and AIS<10, CONTROL2 6 (4-6), p=0.001* (Figure 2).

### Objective evaluation of excessive sleepiness Maintenance of Wakefulness Test (MWT)

- **Sleep latency percentage (Figure 3)**
  **Figure 3:**
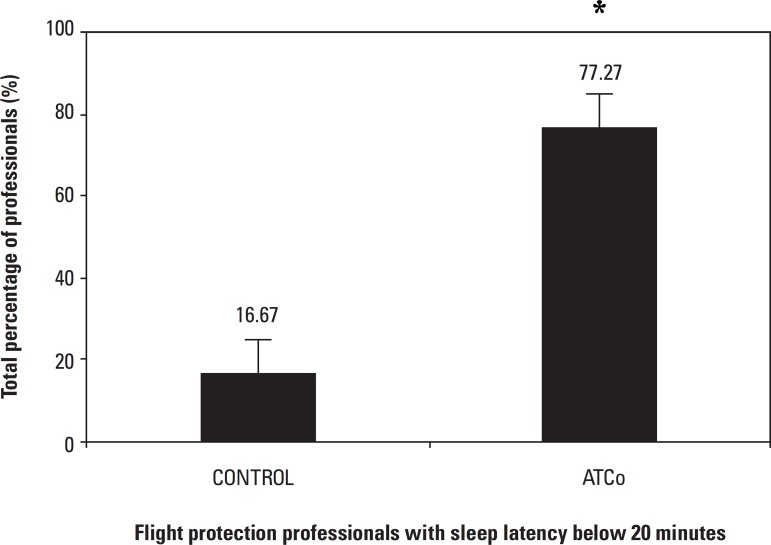
Total percentage of professionals with sleep latency below 20 minutes - Data collected from Air Traffic Controllers. In this graph, the subgroups of AIS were pooled to form one control group. The subgroups of ATCo were also pooled to form a single group. The results found were analyzed by the Mann-Whitney test for objective data, with a significance level of p≤0.05.
  A total of 77.27% of the air traffic controllers (ATCo) and 16.67% of the aeronautical information service operators (AIS), had sleep latency of less than 20 minutes.
- **Sleep latency (Figure 4)**
  **Figure 4:**
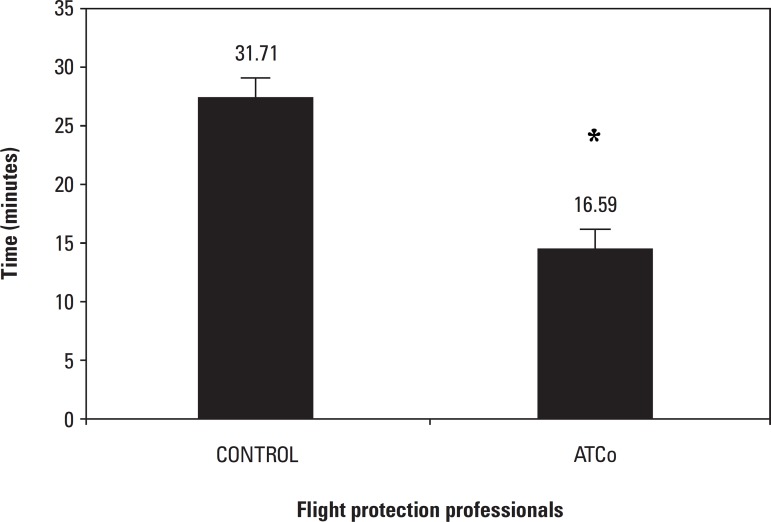
Sleep latency - Data collected among Air Traffic Controllers. The results found were analyzed by the Mann-Whitney test for objective data, with a significance level of p≤0.05.
  The ATCo had sleep latency of 16.59 (3.25-40) minutes, shorter than that of the aeronautical information service operators (AIS) of 31.71(10.63-40) minutes, p<0.05*.

## Discussion

In this study, it was also observed that air traffic controllers had a subjective increase in sleepiness evidenced by the Epworth numerical scale of sleepiness (ESS) while objective evaluation using the maintenance wakefulness test showed a higher percentage of sleep latency of less than 20 minutes.

Sleepiness is a normal biological function defined as an increased probability of falling sleep. However, excessive sleepiness (ES) or hypersomnia, refers to an increased probability of sleeping with a subjective compulsion, and also of taking a nap inadvertently or suffering from sleep attacks when sleep is inappropriate.^13^

The results of this study refer to ES. However, it is important to bear in mind that although this condition occurs many times in the same subject, it is necessary to distinguish ES from the sleepiness present in fatigue, apathy and psychiatric diseases, such as multiple sclerosis, lupus, cancer, infections, Parkinson’s disease, cerebral vascular accident, chronic fatigue syndrome, fibromyalgia, depression, and so forth. Fatigue is defined as tiredness and exhaustion, which is caused by excessive activities, but can be easily remedied by resting. However, ES is often incompletely relieved even with rest or sleep and is usually associated to sleep disorder.^14,15^

This study corroborates the *Statistical Manual of Diagnosis of Mental Disorders - IV* (DSM - IV) that reports shift work as a cause of difficulties in sleep latency and quality, as well as effects such as wakefulness deterioration and excessive sleepiness.^9^

The main causes of ES are chronic sleep deprivation (insufficient sleep), the Obstructive Sleep Apnea-Hypopnea Syndrome (OSAH), narcolepsy, Restless Legs Syndrome/Periodic Limb Movement Disorder (RLS/PLMD), Circadian Rhythm Disorder, drug and medication use, and idiopathic hypersomnia.^16^

Some authors hold there are inter- individual differences in response to acute sleep loss or chronic sleep deprivation, implying that some people may be more resistant than others to the detrimental effects of sleep deprivation on performance.^17^

Inter-individual differences in tolerance for shift work have been studied primarily in terms of external factors affecting alertness on the job or the ability to rest and sleep while at home. However, there is increasing evidence that neurobiological factors also play a role, particularly the major processes involved in the regulation of sleep and wakefulness. These include a sleep homeostatic process seeking to balance wakefulness and sleep and a circadian process seeking to promote wakefulness during the day and sleep during the night. Shift work is associated with a temporal misalignment of these two endogenous processes.^18^

During night work, this misalignment makes it difficult to stay awake during the nightshift and sleep during the day. However, inter-individual variability in the processes involved in sleep/wake regulation is substantial. Recent studies have demonstrated the existence of inter-individual differences in vulnerability to cognitive deficits from sleep loss. Moreover, these inter-individual differences were shown to constitute a trait. Interestingly, self-evaluations of sleepiness did not correlate well with the inter-individual variability trait in objective levels of performance impairment during sleep deprivation. Perhaps because of this discrepancy, in operational settings, the inter-individual differences in vulnerability to sleep loss do not appear to be limited by self-selection mechanisms.^18^ Thus, perhaps further studies are warranted with a larger sample. However, this does not invalidate the credibility and reliability of this study, especially given it is an exploratory investigation, serving as preliminary research to stimulate further exploration.

According to the results of this study, habits related to sleep hygiene such as not using sunglasses when leaving work in the morning after an entire night, sleeping with the lights on, use of stimulants before bed and sedatives to sleep, further contribute to the ES process.

These results corroborate the findings of Åkerstedt (2003) reporting that shift work interferes with sleep, causing sleep disorders and sleepiness. Apart from concerns about flight controller health, there is the issue of flight safety to consider.^15,16^

Since the 1990s, several studies have been conducted emphasizing ES consequences and other impairments of trial and performance related to fatigue, among them, automobile accidents caused by dozing off and disasters.^19^

This type of problem seems to be a global concern because in addition to excessive sleepiness causing other diseases, numerous incidents occur due to lack of proper management of sleep. According to a letter dated 16^th^ of May 2006 from the National Transportation Safety Board (NTSB) to the Federal Aviation Administration emphasizing the importance of sleep management, another incident took place because of flight controller fatigue. Two near collisions on runways at Chicago’s O’Hare International Airport could have been partly caused by over-tired air traffic controllers.^20^

There seems to be a need for discussion with these professionals over irregular shifts that affect their minimum circadian cycle. Some subjects evaluated in this study stated that d working in fixed shifts would be ideal with one team willing to work only in the morning, others in the afternoon and another, only on the night shift.

If it is not possible to organize the rota of the controllers in fixed shifts, then allowing a split in the team to alternate sleep breaks could help in the recovery of sleep latency and vigilance level. A study by Signal et al. (2009) measured sleep during a planned nap on the night shift; and used objective measures of performance and alertness to compare the effects of the nap opportunity versus staying awake. The study subjects were twenty-eight air traffic controllers (mean age 36 years, nine women) and they completed four night shifts (two with early starts and two with late starts). Each type of night shift (early/late start) included a 40-min planned nap opportunity on one occasion and no nap on the other. Polysomnographic data were used to measure sleep and waking alertness [spectral power on electroencephalogram (EEG) during the last hour of the night shift and the occurrence of slow rolling eye movements (SEMs) subsequent to the nap]. A psychomotor performance task was completed at the beginning and end of the shift, and after the nap (or an equivalent time if no nap was taken). Nap sleep resulted in improved psychomotor vigilance task performance, decreased spectral power on EEG and reduced likelihood of SEMs. The occurrence of SWS during the nap decreased spectral power on the EEG. This study indicates that although sleep taken at work is likely to be short and of poor quality it still results in an improvement in objective measures of alertness and performance.^21^

Although this study has been performed using scientific rigor, it remains an exploratory study, paving the way for further research with a larger number of subjects. However, these preliminary findings represent an important contribution to the process of research on sleep disorders in air traffic controllers.
